# Did you get any help? A post-hoc secondary analysis of a randomized controlled trial of psychoeducation for patients with antisocial personality disorder in outpatient substance abuse treatment programs

**DOI:** 10.1186/s12888-016-1165-2

**Published:** 2017-01-09

**Authors:** Birgitte Thylstrup, Sidsel Schrøder, Mats Fridell, Morten Hesse

**Affiliations:** 1 Aarhus University, Centre for Alcohol and Drug Research, Aarhus, Denmark; 2 Department of Psychology, Lund University, Lund, Sweden

**Keywords:** Antisocial personality disorder, Substance use disorder, Perceived help, Randomized trial, Impulsive lifestyle counselling

## Abstract

**Background:**

People in treatment for substance use disorder commonly have comorbid personality disorders, including antisocial personality disorder. Little is known about treatments that specifically address comorbid antisocial personality disorder.

**Methods:**

Self-rated help received for antisocial personality disorder was assessed during follow-ups at 3, 9 and 15 months post-randomization of a randomized trial of psychoeducation for people with comorbid substance use and antisocial personality disorder (*n* = 175).

**Results:**

Randomization to psychoeducation was associated with increased perceived help for antisocial personality disorder. Perceived help for antisocial personality disorder was in turn associated with more days abstinent and higher treatment satisfaction at the 3-month follow-up, and reduced risk of dropping out of treatment after the 3-month follow-up, and perceived help mediated the effects of random assignment on days abstinent at 3-month.follow-up.

**Conclusions:**

Brief psychoeducation for antisocial personality disorder increased patients’ self-rated help for antisocial personality disorder in substance abuse treatment, and reporting having received help for antisocial personality disorder was in turn associated with better short-term outcomes, e.g., days abstinent, dropout from treatment and treatment satisfaction.

**Trial registration:**

ISRCTN registry, ISRCTN67266318, retrospectively registered 17/7/2012.

**Electronic supplementary material:**

The online version of this article (doi:10.1186/s12888-016-1165-2) contains supplementary material, which is available to authorized users.

## Background

Common mental health problems like depression and anxiety often co-occur with problematic alcohol and drug use [1–3], as do personality disorders, in particular antisocial personality disorder [ASPD] [4, 5]. These comorbid conditions are associated with lower quality of life for patients [6], and may also interfere with treatment or with social rehabilitation [7, 8].

There is now increasing evidence that providing onsite treatment in community substance abuse treatment that targets comorbid conditions can benefit patients in a range of settings, and that this applies to both substance use and symptoms of anxiety and depression [9–12].

However, given that ASPD is one of the most common comorbidities in illicit drug use disorder [DUD], it is surprising that so little research has been conducted to assess ways to address ASPD in substance use disorder [SUD] treatment settings, or to improve the outcomes for patients with comorbid ASPD. This is especially surprising, since ASPD may have an impact on the completion of treatment, although the impact depends on the context surrounding the treatment episode, i.e., patients with ASPD drop out when they have no tangible rewards to gain from treatments [13]. When patients do not have any tangible rewards to gain from being in treatment, the challenge for clinicians is to help patients see that treatment has relevance for them.

### Impulsive lifestyle counselling

One intervention designed to improve the outcomes of treatment for SUD in patients with ASPD, the Impulsive Lifestyle Counselling [ILC] program [14], was developed and tested in a pragmatic randomized trial. The trial found that ILC had significant effects on days abstinent from substance use and severity of drug use at the 3-month follow-up, and that aggression declined in both treatment as usual and ILC with no significant differences between interventions [15], and that the ILC program had significant effects on the risk of dropout from substance abuse treatment [16]. However, one question that has not been previously assessed in this study is the perceived helpfulness of treatments for mental health comorbidity. In general population studies, the perceived helpfulness of treatments for depression is sometimes assessed, usually through Likert scale items (e.g. [17, 18]), but research is scarce. Examining perceived helpfulness of treatment offers is important, as individual attitudes and beliefs about treatment can influence future service use and treatment seeking behavior [19]. For example, previous studies show that perceiving treatment as ineffective is one of the commonly cited barriers deterring individuals from seeking further treatment [20]. Perceived helpfulness may be even more important for people with ASPD, since patients with ASPD are prone to anger, and often find themselves in conflicts with others, including clinic staff [21]. Patients with any psychiatric disorder who enter treatment have to face considerable challenges to engage and remain in treatment. Usually, patients with psychiatric disorders have lived a life with critical or concerned family members, while at the same time experiencing a substantial degree of self-criticism. Enrolling in treatment often involves further challenges to the individual patient’s belief systems. In addition, treatment is likely to focus on the need to change coping strategies, which may be perceived by the patient as a form of help for dealing with the present, but may be ineffective in the long-term [22]. In this context, people who are involved with drugs and crime often have strong social bonds with others who use drugs and break the law, and may have value systems that conflict with collaborating with anyone who can be regarded as being authority figures, both factors which further complicate the treatment process [23]. At the same time, patients with DUD need to contemplate getting by without the drugs that have not only helped them cope with daily hassles, and which have also become a central part of their identity. As a consequence, entering into treatment becomes a difficult transition that requires a substantial amount of confidence in treatment; supporting that confidence is a key task for the treatment providers. Feeling that treatment is relevant and helpful may give the patients motivation to engage more in treatment and cooperate better with clinicians.

#### Aims

The aims of this study were to assess perceived help for ASPD using secondary analyses based on a randomized clinical trial of six sessions of psychoeducation for comorbid SUD and ASPD [15, 16]. We predicted that randomization to the psychoeducational program would increase the perception of having received help for ASPD as part of the substance abuse treatment during the study period. Finally, a third aim was to assess whether self-rated help for ASPD mediated the effects of the ILC program in this sample.

## Methods

### Study design for the ILC study

The ILC study was a Phase I pragmatic randomized controlled trial with single blind assessments that was carried out at community-based outpatient substance abuse treatment clinics in Denmark between January 2012 and July 2014. The details of the study are reported in two previous reports [15, 16]. Inclusion criteria to the study were: between 18 and 65 years old; met criteria for ASPD using the Mini International Neuropsychiatric Interview [24]; and able to provide written informed consent; seeking treatment or already in treatment for a SUD.

#### Ethics, consent and permissions

The present project was reviewed by the regional ethics committee of the Capital Region of Denmark and deemed exempt from a formal evaluation (J#H-3-2012-FSP45). The trial was registered in the ISRCTN register (#ISRCTN67266318). All participants gave written and verbal consent to participation and to be contacted for follow-up interviews.

### Recruitment and randomization

Study participants were identified by clinicians at the participating sites from new and existing patients receiving outpatient community treatment for a SUD. After agreeing to be contacted, a trained clinician at each site invited potential participants to take part in a screening to assess the diagnosis of ASPD. Those who met inclusion criteria were told that their responses indicated ASPD, and the clinician would then review their responses to the ASPD MINI module with the patients, and ask if they were willing to talk to a trained ILC counsellor about it. Patients who agreed to see a counsellor and who provided written informed consent, subsequently completed the baseline assessment and were randomly allocated to either one of two treatment groups: treatment as usual (TAU) or the Impulsive Lifestyle Counselling (ILC).

Randomization was stratified by clinic. The randomization schedules were generated by the trial coordinator and kept secure and confidential by the trial coordinator at the study coordinating center in Copenhagen. The trial coordinator informed the referring clinician of the result of randomization immediately after being notified that the patient had been assessed and found to be eligible for study participation. Following this, the clinician informed the patient of the result. In cases in which the patients were randomized to the ILC program, the clinician contacted one of the ILC counsellors at the uptake unit with the participants’ details so that the program sessions could be initiated immediately after randomization.

Because the randomization took place immediately after the screening interview, the trial coordinator was unable to check whether the baseline assessment was complete before randomizing, and patients with incomplete data at baseline were excluded after randomization.

### Treatment conditions

#### Treatment as usual (TAU)

All participants received whichever form of treatment they would have received at the participating treatment service if the trial had not taken place. Treatment always included access to opioid substitution treatment for patients who needed it, either with methadone, buprenorphine or a combination of methadone and injectable diacetylmorphine; psychosocial support in the form of casework and counselling; and referral to residential rehabilitation for patients who needed it. At some clinics, a liaison psychiatrist would see the patients onsite, whereas patients in other clinics would be referred to an off-site psychiatrist for diagnosis and treatment of other psychiatric conditions, such as attention-deficit/hyperactivity disorder, anxiety or depression.

#### Impulsive Lifestyle Counselling (ILC)

In addition to the services available to the patients who received TAU, patients randomized to ILC were offered up to six one-hour ILC sessions. The ILC program is a highly structured workbook on psychoeducational intervention for people with ASPD [14]. The form and content of the sessions were adapted from the manual for the Lifestyle Issues Program [25], and in line with the Lifestyle Issues Program, the main objective of the ILC program is to support the individual patient in awareness raising, in taking responsibility for addressing behavioral problems, and in opening up to the possibility of a change of lifestyle. Further, similar to the psychoeducational approach by Banerjee and colleagues, the psychoeducational approach in the ILC is intended to function as an educative and collaborative exercise that can improve patient engagement in further treatment [26, 27].

The first five sessions takes place once a week; each session covers a specific topic, and includes specific questions that the counsellor asks, as well as pre-printed handouts and worksheets given to the patient. The first session focuses on the objective of the ILC program, and on identifying thoughts and behaviors related to ASPD. The second session is based on an adapted version of the Antecedents-Beliefs-Consequences model from Rational-Emotive Behavior Therapy [28], linking the patients’ impulsive behaviors to the immediate consequences. Session 3 focuses on how impulsive and destructive behaviors are related to specific value systems and beliefs related to ASPD, and Session 4 presents the concept of values and discusses what values may support or prevent the patient in change of lifestyle. Session 5 focuses on the patient’s social networks and how social contacts may support or challenge lifestyle changes. The last session is a booster session that takes place 6 weeks after Session 5, at which the patient is invited to talk about the topics from the previous five sessions that he or she finds most relevant for future work with lifestyle changes.

Like the Lifestyles Issues Program, the ILC program is designed so that no prior professional training or special facilities of any sort are necessary in order for the intervention to be feasible in most clinical settings where people with DUD receive treatment. However, prior to delivering the study intervention, all counsellors participated in a one and a half day workshop to practice the workbook and discuss issues relating to treating people with ASPD in general. All counsellors were required to both keep written records and make audio recordings of the sessions.

### Follow-up procedures

For the three follow-up waves at 3, 9 and 15 months after randomization, patients were contacted first through any phone number they had provided. If it was not possible to establish contact using this procedure, the patient was contacted through the clinic at which he or she had been screened for study participation. If a patient still could not be reached, researchers would ask his or her case manager at the study clinic if they could set up a time for a meeting, or when it would be feasible to meet the patient. Patients who could still not be reached this way were contacted through the contact information that they had provided to significant others, and finally through other accessible services (e.g. prison or psychiatry). In a few cases, the patients were eventually located through the Central Personal Register. Once a patient had been located, and if it was possible to speak with the patient directly, a place and time for an interview was scheduled. If the patient did not show up for a face-to-face interview, a new time would be scheduled, and only after several failed attempts was a telephone interview suggested. If the patients stated that they were not willing to be interviewed, they would be asked if they would agree to be contacted at a later point, and if they refused, they were not contacted again.

#### Measures

The primary outcome variable for the present study was the degree to which respondents reported having received help for ASPD. In this article, we refer to this as “self-rated help for ASPD”.

In order to assess perceived help, two Likert scale questions were asked. First, respondents were asked if they believed they had, or had had, ASPD while they were in treatment, with response options 3 = definitely, 2 = probably, 1 = probably not, and 0 = not at all. Interviewers were instructed to refer to the MINI adult ASPD items which were included on the questionnaire. Perceived help for each disorder was measured by asking respondents if they had received any form of help for the ASPD during the course of SUD treatment. Here response options were 3 = having received help to a very high degree, 2 = having received help to some degree, 1 = having received a little help, and 0 = not having received help.

#### Diagnosis of ASPD

The Mini International Neuropsychiatric Interview [MINI] was used to screen for ASPD [29, 30]. The MINI was designed to assess DSM-IV and ICD-10 diagnoses [29, 30], and is a fully structured, brief and valid diagnostic interview that can be conducted by a lay person, and is well accepted by patients [31]. The ASPD module consists of six questions concerning childhood conduct disorder and six questions about adult ASPD. Previous research indicates that the MINI module for ASPD identifies prison inmates with more serious mental health problems, more substance abuse problems and a more serious and chronic history of offending behavior as compared with other inmates [32–34], and that ASPD diagnosed according to the MINI is associated with illicit drug use in the general population [35].

Additional demographic data were collected on a separate sheet, including education, employment history and history of homelessness, residential treatment for SUD, incarceration and psychiatric hospitalizations.

Current substance use severity was measured using the drug use composite score from the Addiction Severity Index (ASI), of which has demonstrated high concordance with DSM-IV SUDs [36].

Internal consistency for the drugs composite score in this sample was α = 0.60 at baseline, α = 0.60 at the 3-month follow-up, and α = 0.64 at the 9-month follow-up. A second outcome measure was days abstinent in the past 30 days. All substance use data were collected at baseline and at each follow-up wave.

General aggression was measured using the 12-item version of the Buss-Perry Aggression Questionnaire (BPAQ, [37]), a commonly used measure of general aggression in both general population and forensic samples with good psychometric properties. Sample items include statements such as: “Given enough provocation, I may hit another person.” and “I often find myself disagreeing with people.” The items are scored on a five-point Likert scale ranging from 1 (“extremely uncharacteristic of me”) to 5 (“extremely characteristic of me).” Sample internal consistency for the BPAQ was α = 0.82 at baseline, α = 0.81 at the 3-month follow-up, α = 0.80 at the 9-month follow-up, and α = 0.81 at the 15-month follow-up.

Interpersonal aggression was measured using the 14-item version of the Self-Report of Aggression and Social Behavior Measure (SRASBM, [38]) a measure of interpersonal aggressive acts and dispositions. Sample items include statements such as: “My friends know that I will think less of them if they do not do what I want them to do.” and “When I am mad at a person, I try to make sure she/he is excluded from group activities (such as going to the movies or to a bar).” Items are rated on a five-point Likert scale from 0 (“Never”) to 4 (“Very often”). The internal consistency for the SRASBM in this sample was α = 0.78 at baseline, α = 0.81 at the 3-month follow-up, α = 0.82 at the 9-month follow-up, and α = 0.82 at the 15-month follow-up.

Treatment satisfaction was assessed using the 8-item client satisfaction questionnaire (CSQ, [39]). The internal consistency for the CSQ was α = 0.90 at baseline, α = 0.94 at the 3-month follow-up, α = 0.95 at the 9-month follow-up, and α = 0.96 at the 15-month follow-up.

### Blinding

Research technicians not affiliated with the study clinics carried out all assessments at the follow-up interviews and were blind to treatment group allocation.

### Analyses

Random-effects regression was used assess to the likelihood of a high versus low endorsement of having received help for ASPD during the three follow-up waves. Random effects were estimated for both patient and study site, and covariates were gender, age, receiving substitution treatment at baseline, treatment satisfaction at baseline and assessment wave (3 months being the reference category, and 9 and 15 months being the dummy codes).

The study applied intent-to-treat analyses, i.e. data were analyzed by randomization arm irrespective of attendance or treatment compliance. Covariates were gender, age, receiving substitution treatment at baseline, and assessment wave (3 months being reference, 9 and 15 months being dummy codes). The predictor was randomization status.

Following this, random-effects regression analysis was conducted for each of the primary outcomes of the trial, to test whether self-rated treatment for ASPD was associated with improved outcomes for the trial. In each of these analyses, the predictors were: perceived help for ASPD, age, gender, substitution at baseline and the baseline value for the outcome of interest, and random effects were estimated for study site and patient.

An identical random effects regression was conducted for global treatment satisfaction, defined in the trial registration as a secondary outcome. Survival analysis was used to assess whether perceived help for ASPD was associated with risk of subsequent dropout from the 3-month follow-up using Cox proportional hazards regression with clustering for site, again adjusted for gender, age and substitution treatment at baseline. Of the 128 patients who were interviewed at the 3-month follow-up, 18 had been discharged from treatment, and were thus no longer at risk of dropping out. Therefore, the survival analysis was conducted using the remaining 110 patients.

Finally, we intended to test whether self-rated help for ASPD mediated the effects of the ILC program on our two significant outcomes, days abstinent and ASI drugs composite score, we conducted two mediation analyses. First, we assessed whether effects of the ILC program on self-rated help for ASPD mediated the effects of ILC on days abstinent, and then we assessed whether the effects of the ILC program on self-rated help for ASPD mediated the effects of ILC on the drugs composite score [15]. In mediation analysis, some of the effect of the independent variable, the IV, is transmitted to the dependent variable, the DV, through the mediator variable, the MV and some of the effect of the IV passes directly to the DV. Mediation occurs when the following four statements are true: (1) the IV has significant effects on the DV; (2) the IV has significant effects on the MV; (3) the MV has significant effects on the DV; and (4) once the effects of the MV on the DV are controlled, the effect of the IV on the DV is significantly reduced [40]. In the mediation analyses, the IV was randomization status (ILC or TAU), the MV was self-rated help, and the DV was days abstinent in the first analysis, and the ASI drugs composite score in the second. In both mediation analyses, baseline values for the DV, substitution treatment, age and gender were included as control variables. We present the results of a power analysis to detect a significant mediation effect based on Thoemmes, Mackinnon & Reiser [41]. The presented analysis is based on the observed effects in this study, and was adapted from Thoemmes and colleagues’ work using MPlus 7.4. Specifically, we retained the a and b coefficients (i.e., the effect of ILC on perceived help and the effect, and the effect of perceived help on days abstinence), as these two are the only ones that are of consequence for the power analysis [42].

To assess the mediation effect, we used the Preacher and Hayes method. The indirect effect was obtained with 5,000 bootstrap resamples [43]. The mediation analyses were conducted using only when both the IV (random assignment) and the MV (self-rated help for ASPD) had significant on the outcome of interest, as mediation cannot occur when either the direct effect of the IV or the MV is not statistically significant.

## Results

### Participants

Characteristics of the patients randomized to each of the two conditions are summarized in Additional file 1: Table S1. The 175 participants were 156 men and 19 women, the mean age was 32.4 years of age (standard deviation [SD] = 9.0), and 38.3% were receiving opioid substitution treatment at the time of randomization.

### Follow-up rate

Of 175 patients randomized, 130 were interviewed at 3 months (74%), 118 at 9 months (67%), and 107 at 15 months (61%). Of these, a small number of respondents (ranging from 2 to 12 respondents at each follow-up wave), did not respond to the question about self-rated help for ASPD. The main reason that respondents did not answer the item was that the informant had to be interviewed by telephone, and in the telephone interviews, we asked only about the primary outcomes for this study. When patients had not responded to an item at a given follow-up wave, they were excluded from the analysis of that variable at that point.

### Perception of having received help for ASPD

**Fig. 1:**
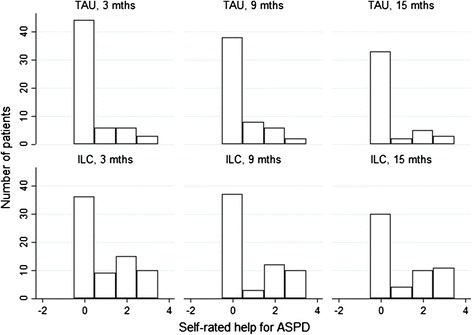
Self-rated help for ASPD. Response categories. 3 = having received help to a very high degree, 2 = having received help to some degree, 1 = having received a little help, and 0 = not having received help

The ratings of help for ASPD are summarized in Fig. 1. As can be seen in Fig.1, regardless of randomization assignment, a very large proportion of patients reported that they did not receive any help for ASPD at all.

The mixed effects regression is summarized in Table 1. The primary aim of this analysis was to test whether the intervention had a direct effect on self-rated help. Higher scores on help for ASPD were associated with randomization to ILC (*p* < .001), and older patients gave lower scores on having received help for ASPD (*p* = .016). No other covariates were significant. The intraclass correlation for site was small (ρ = .082, 95% CI = .015–0.337), and the intraclass correlation for patient was moderate (ρ = .415, 95% CI = .295–0.546).

**Table 1:** Predictors of self-rated help for antisocial personality disorder (*n* = 149)

	Coefficient	*P*-value
Randomized to ILC	0.454 (0.195–0.713)	.001
Conduct disorder item count	0.083 (-0.096–0.262)	.363
Adult ASPD item count	0.136 (-0.005–0.277)	.059
Assessment wave		
9 months	−0.042 (-0.256–0.173)	.703
15 months	0.037 (-0.189–0.263)	.746
Female gender	−0.073 (-0.383–0.337)	.726
Age	−0.014 (-0.031–0.002)	.091
Treatment satisfaction at baseline	0.125 (-0.099–0.341)	.254
Intraclass correlation		
Site	.116 (.031**–**.349)	
Patient	.377 (.248**–**.527)	

### Associations between self-reported help and primary outcomes

The results of mixed regression analyses for self-reported outcomes are summarized in Table 2. The results of these analyses were to test whether self-rated help was associated with better outcomes.

**Table 2:** Associations between self-rated help for ASPD and outcomes (*N* = 149)

	Days abstinent	Drugs composite	Alcohol composite	BPAQ	SRASBM	Treatment satisfaction
Help for ASPD	**1.216 (0.161–2.271)**	0.004 (-0.006**–**0.015)	−0.001 (-0.017**–**0.014)	**0.103 (0.012–0.194)**	0.024 (-0.014**–**0.062)	**0.077 (0.016–0.140)**
Baseline value	**0.383 (0.245–0.522)**	**0.204 (0.082–0.325)**	**0.485 (0.398–0.571)**	**0.582 (0.481–0.684)**	**0.341 (0.255–0.423)**	**0.692 (0.522–0.862)**
Substitution	**−5.969 (-9.486–-2.453)**	**0.109 (0.075–0.143)**	0.012 (-0.031**–**0.055)	0.226 (-0.035**–**0.487)	0.082 (-0.040**–**0.205)	−0.150 (-0.382**–**0.083)
Female gender	Ref.	Ref.	Ref.	Ref.	Ref.	Ref.
Male gender	0.135 (-4.211**–**4.481)	0.012 (-0.028**–**0.519)	0.009 (-0.045**–**0.064)	−0.207 (-0.541**–**0.128)	−0.149 (-0.008**–**0.005)	0.034 (-0.251**–**0.319)
Age	**0.233 (0.048–0.418)**	−0.000 (-0.002**–**0.002)	0.001 (-0.001**–**0.003)	−0.003 (-0.017**–**0.011)	−0.001 (-0.008**–**0.005)	−0.000 (-0.012**–**0.012)
3-month follow-up	Ref.	Ref.	Ref.	Ref.	Ref.	Ref.
9-month follow-up	**2.328 (0.307–4.349)**	**−0.038 (-0.058–-0.017)**	0.000 (-0.032**–**0.032)	**−0.346 (-0.531–0.161)**	**−0.095 (-0.166–-0.023)**	−0.055 (-0.168**–**0.058)
15-month follow-up	1.970 (-0.150**–**4.089)	−0.044 (-0.066**–**-0.023)	−0.024 (-0.057**–**0.009)	**−0.433 (-0.627–-0.238)**	**−0.130 (-0.206–-0.054)**	−0.072 (-0.190**–**0.047)
Intraclass correlation						
Site	.000	.000	.000	.000	.000	.000
Patient	.509 (.404**–**.612)	.043 (.321**–**.551	.307 (0.198**–**0.442)	.360 (.252**–**.484)	.502 (.377.**–**.626)	.630 (.534**–**.718)

*Notes*

*ASPD* antisocial personality disorder

Coefficients significant at *p* < .05 are in boldface

Perceived help for ASPD was associated with more days abstinent (B = 1.29, z = 2.32, *p* = .020), treatment satisfaction (B = 0.079, z = 2.50, *p* = .012), and self-reported general aggression on the BPAQ (B = 0.103, z = 2.13, *p* = .033). Associations with ASI drugs composite score (B = 0.004, z = 0.81, *p* = .416), B = -0.002, z = -0.30, *p* = .766), and interpersonal aggression (B = 0.027, z = 1.38, *p* = .169) were not statistically significant.

### Association with secondary outcomes

Self-reported treatment for ASPD was associated with higher satisfaction with treatment (B = 0.077, *p* = .015). The survival analysis was significant (Wald *χ* ^2^(4) = 13.83, *p* = .008), and self-reported treatment for ASPD at the 3-month follow-up was associated with lower risk of subsequent dropout (Hazard ratio [HR] = 0.762, z = -2.27, *p* = .023). In addition, age was negatively associated with dropout (HR = 0.935, z = -2.02, *p* = .044) and substitution treatment at baseline was negatively associated with subsequent dropout (HR = 0.464, z = 2.08, *p* = .037).

### Mediation analyses

**Fig. 2:**
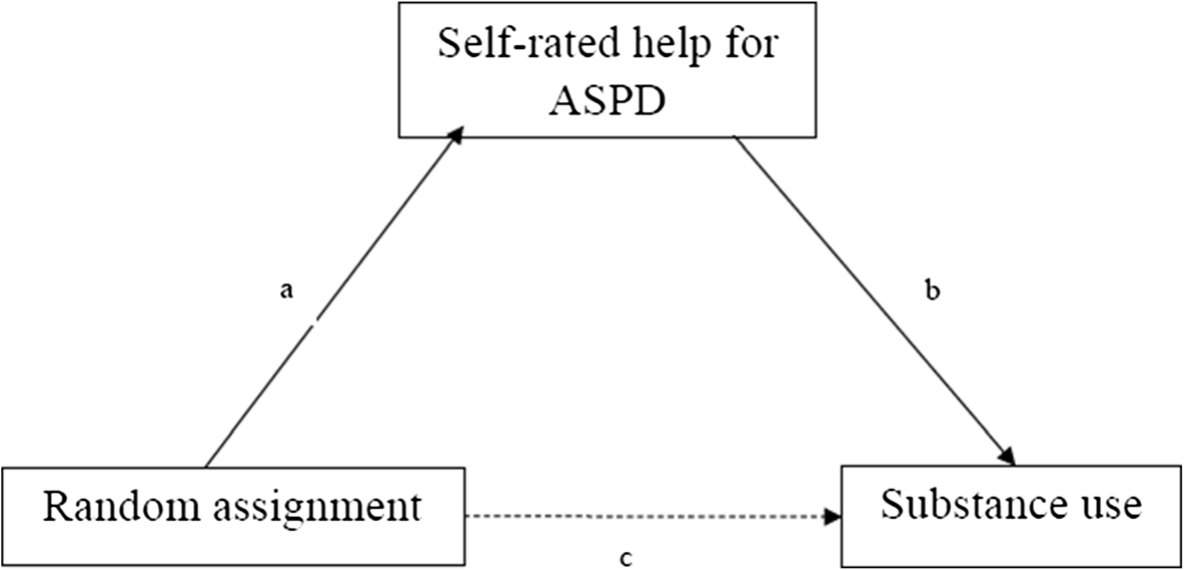
Theoretical mediation model

The theoretical mediation analyses are summarized Fig. 2. Following the convention in mediation analysis, the *a* coefficient is the effect of the IV, ILC, on the MV, perceived help for ASPD. The *b* coefficient is the effect on the MV on the DV, days abstinent or drugs composite score. The *c* coefficient is the direct effect of the IV on the DV.

Given that only days abstinent satisfied the two first criteria for mediation (direct effects of random assignment and effect of MV on DV), we only analyzed mediation for this variable. The results are summarized in Table 3. Of the 128 patients who answered the question about help for ASPD, four had not answered the items about days abstinent at baseline, and had to be excluded, leaving 124 patients for this analysis. In the first step, days abstinent at the 3-month follow-up was associated with randomization to ILC (Z = 2.06, *p* = .042). In the second step, perceived help was associated with randomization to ILC (Z = 2.85, *p* = .005). In the third step, perceived help was associated with more days abstinent (Z = 2.17, *p* = .032), and randomization to ILC was no longer significantly associated with days abstinent (Z = 1.47, *p* = .144). For days abstinent, 27% of the effect of randomization was explained by self-rated help for ASPD (observed indirect coefficient = 1.037, bootstrap CI 0.050–2.600) and given that the CI does not overlap with zero, the indirect effect was significant [43].

**Table 3:** Summary of mediation analysis

	Days abstinent (*n* = 124)		
	Coefficient	T-score	*P*-value
a coefficient	0.53	2.85	.005
b coeffient	1.96	2.17	.032
c coefficient without mediator	3.81	2.06	.042
c coefficient with mediator	2.77	1.74	.144
Preacher and Hayes indirect effect	1.037 (0.050–2.600)		
		Z-score	
Sobel	1.04	1.73	.084

### Power to detect mediation effects

A post-hoc power analysis was conducted to assess mediation effects for the present study based on the recommendations of Thoemmes, Mackinnon & Reiser [41]. The analysis was based on the Mplus code for a single mediator, and we substituted the values from the present study for the values in their code. The analysis showed that the indirect effect would be significant in 85.3% of replications with a sample of 124 patients. With 90 patients, 62.9% of tests would be significant, with 110 patients, 78.1% would be significant, ant with 140 patients, 91.4% would be significant. The Mplus output is in Additional file 2.

## Discussion

This study had three, related aims. First: to test if the ILC program had an effect on perceived help for ASPD during treatment. In line with the predictions, randomization to the ILC program did increase the endorsement of the perception of having received help for ASPD while in treatment for a SUD. This is an important finding, because it means that by offering brief psychoeducation, in this case the ILC program, to patients with ASPD, it is possible to increase the likelihood that they will feel that treatment addresses a significant problem in their life.

In light of the fact that the intervention had an impact on retention and abstinence as reported in previous articles, it is very encouraging that patient perceptions converged with other findings in showing that the ILC program increased the degree to which the SUD treatment was perceived as helpful by the patients.

The second aim was to test if perceived helpfulness was associated with better outcomes, adjusting for baseline values. The findings concerning this aim were less consistent: perceived help was associated with more days abstinent, higher treatment satisfaction and decreased risk of dropping out of treatment, but not with drug severity, or self-reported interpersonal aggression. The link between perceived help and outcomes may be important, even when this has nothing to do with the intervention under study in this trial. If perceived help is important for patients with ASPD, other interventions that increase perceived help may be useful in improving outcomes for patients with ASPD.

However, one outcome was negatively associated with self-rated help: patients who rated higher on having received help for ASPD reported more general aggression at follow-up waves on the BPAQ. This finding may seem paradoxical, as it is somewhat counterintuitive that someone who has received more help for ASPD would be more aggressive, while at the same time being more satisfied with treatment and less likely to drop out of treatment. One possible explanation is that patients who remember having discussed issues related to offending, impulsive and violent behavior may be more aware their own aggression, or more willing to disclose aggressive thoughts and behaviors to an interviewer.

In relation to the third aim, we were able to confirm that perceived help for ASPD mediated the effects of our intervention on only one of our primary outcomes. Our study was not powered to test such an association a priori, as the power analysis was conducted simply to assess the likelihood of finding a significant effect of the intervention [15]. The post hoc power analysis suggested the study did have the power to detect a mediation effect. In order to have a reasonable likelihood of detecting a significant mediation effect, the a and b paths must both be of considerable size [41]. Indeed, in the present study, the effect of random assignment on perceived help (i.e., the a coefficient) and the association between perceived help and days abstinent (i.e., the b coefficient), were strong. However, this must be interpreted with great caution, as it is possible that third variables confounded the mediator and the dependent variable, a situation which could bias the estimate of the indirect effect [44]. Clearly, larger trials are needed to address potential mechanisms of action, including mediation through perceived help.

Another important finding from this study is that among the patients included in this trial, whenever an interviewer asked them during the three follow-up waves whether they had received help, a majority indicated that they had received little if any kind of help for ASPD during their treatment, regardless of whether they were randomized to ILC or TAU. This is important, because it indicates that being in treatment for a SUD in itself was rarely perceived as help for ASPD. Also, while the brief psychoeducational intervention increased the likelihood that a patient would report help for ASPD slightly, it did not change the perception of the majority of patients, and much more work is needed to ensure that a substantial proportion of patients with ASPD get treatment.

Whether the statistically significant effect of ILC on perceived help should be interpreted as patients perceiving the brief psychoeducational program as helpful in itself, or that it changed the way in which patients evaluated their overall SUD treatment, cannot be addressed based on the available data. Our questions did not ask about the ILC program in relation to receiving help for ASPD, only if they had received any help for ASPD as part of their overall treatment.

A number of limitations must be acknowledged for this study. First, the questions used to assess self-rated ASPD and self-rated help for ASPD were not validated prior to inclusion in this study. We are, however, aware of no validated instruments to assess these issues, and the question used to assess self-rated help was modelled after questions used in epidemiological research. Secondly, although the study is not small in comparison to other randomized studies of comorbid conditions in populations with SUD, the sample size was too small to address some very crucial research questions, such as whether self-rated helpfulness mediated the link between randomization status and self-rated ASPD.

Another substantial limitation is that we were able to maintain only a small minority of our patients in psychoeducation long enough to receive the full dose of psychoeducation. Although only a few patients dropped completely out of SUD treatment during the period where they were meant to receive the psychoeducational intervention, many missed appointments repeatedly or stopped showing up altogether, so that they did not receive an optimal amount of psychoeducation. This is a significant limitation of the trial in general, and this trial has certainly not exhausted the ways to improve services for people with ASPD.

## Conclusions

Brief psychoeducation for antisocial personality disorder increased patients’ self-rated help for antisocial personality disorder in substance abuse treatment, and patients’ self-reporting of having received help for antisocial personality disorder was in turn associated with better short-term outcomes. Future research should maintain a focus on the role of perceived help in the process and outcomes of treatment, across various types of comorbid conditions in patients with substance use disorders.

## Additional files

Additional file 1: Table S1. Descriptive data at baseline for control and intervention group. (DOCX 13 kb)
Additional file 2: Mplus output for power analysis for mediation. (DOCX 16 kb)

## Abbreviations

ASI: Addiction severity index
ASPD: Antisocial personality disorder
BPAQ: Buss-Perry aggression questionnaire
CSQ: Client satisfaction questionnaire
DUD: Drug use disorder
DV: Dependent variable
HR: Hazard ratio
ILC: Impulsive lifestyle counselling
IV: Independent variable
MINI: The Mini International Neuropsychiatric Interview
MV: Mediating variable
SD: Standard deviation
SUD: Substance use disorder
SRASBM: Self-report of aggression and social behavior measure
TAU: Treatment as usual
