# Supplementary material for: Did you get any help? A post-hoc secondary analysis of a randomized controlled trial of psychoeducation for patients with antisocial personality disorder in outpatient substance abuse treatment programs
Source: BMC Psychiatry. 2017 Jan 9;17:7. doi: 10.1186/s12888-016-1165-2 (PMC5223491; doi:10.1186/s12888-016-1165-2)
Supplement: Additional file 1: Table S1. — Descriptive data at baseline for control and intervention group. (DOCX 13 kb) [file 12888_2016_1165_MOESM1_ESM.docx]

**Table S1** Baseline characteristics by randomization status.

|  | Treatment as usual (n = 80) | Impulsive lifestyle counseling (n = 95) | p-Value |
| --- | --- | --- | --- |
| Male gender | 90.0% | 88.4% | .811 |
| Female gender | 10.0% | 11.6% |  |
| Age | 33.19(8.65) | 31.67(9.18) | .265 |
| Previous treatment | 61.3% | 60.0% | .877 |
| Opioid substitution treatment | 38.8% | 37.9% | 1.000 |
| Methadone treatment | 25.7% | 28.3% | .729 |
| Buprenorphine treatment | 9.5% | 8.7% | 1.000 |
| History of incarceration^a^ | 56.2% | 54.8% | .876 |
| History of psychiatric hospitalization | 30.6% | 26.9% | .607 |
| History of homelessness^b^ | 58.3% | 46.2% | .157 |
| Received welfare benefit in past 30 days | 80.8% | 79.4% | .847 |
| Treatment satisfaction | 2.26(0.58) | 2.15(0.65) | .301 |
| ASI drug use Composite score^c^ | 0.19(0.12) | 0.20(0.13) | .483 |
| ASI alcohol composite score^c^ | 0.15(0.22) | 0.15(0.22) | .996 |
| Days of substance use |  |  |  |
| Days of any alcohol use | 5.76(8.02) | 5.33(7.30) | .725 |
| Days of five or more alcoholic drinks | 3.07(5.53) | 3.40(5.63) | .710 |
| Heroin | 1.37(4.81) | 0.53(2.68) | .164 |
| Methadone | 7.36(12.92) | 7.68(12.90) | .874 |
| Other opioids | 0.86(3.95) | 0.84(3.12) | .980 |
| Tranquilizers and sedatives | 7.41(11.94) | 6.68(11.58) | .748 |
| Cocaine | 1.49(4.10) | 1.61(4.61) | .864 |
| Amphetamine | 0.34(0.83) | 1.39(3.64) | .019 |
| Cannabis | 13.0(12.96) | 14.2(12.97) | .579 |
| Hallucinogens | 0.11(0.65) | 0.03(0.23) | .269 |
| Inhalants | 0.00(0.00) | 0.02(0.15) | .211 |
| Buprenorphine | 2.34(7.80) | 1.33(6.06) | .368 |
| Poly-substance | 6.38(10.39) | 7.51(11.36) | .524 |

Notes: Values are percentages or means with standard deviations in parentheses.

p-Values are Fisher's exact for χ2 tests and t-test for continuous variables.

ASI: Addiction Severity Index.

a Missing data for 9 patients.

b Missing data for 10 patients.

c Missing data for 8 patients.
