# Supplementary material for: Did you get any help? A post-hoc secondary analysis of a randomized controlled trial of psychoeducation for patients with antisocial personality disorder in outpatient substance abuse treatment programs
Source: BMC Psychiatry. 2017 Jan 9;17:7. doi: 10.1186/s12888-016-1165-2 (PMC5223491; doi:10.1186/s12888-016-1165-2)
Supplement: Additional file 2: — Mplus output for power analysis for mediation. (DOCX 16 kb) [file 12888_2016_1165_MOESM2_ESM.docx]

Mplus VERSION 7.4

MUTHEN & MUTHEN

10/31/2016 1:29 PM

INPUT INSTRUCTIONS

TITLE: IMPULSIVE LIFESTYLE COUNSELLING TO PERCEIVED HELP FOR ASPD - MEDIATION PATH MODEL

MONTECARLO:

names are ILC HELP PDA; !Define variable names;

cutpoints = ILC (0); !Define cutpoint to generate binary treatment

nobs = 124; !Power is estimated for this sample size;

nreps = 10000; !Number of replications;

seed = 2222; !Seed value for random number generator;

ANALYSIS: TYPE=meanstructure;

MODEL POPULATION:

[ILC @ 0]; !mean of ILC set to 0;

ILC @ .25; !total variance of ILC set to .25;

[HELP @ 0.744]; !mean of help set to 0 as in original;

[PDA @ 11.822]; !mean of pda set to 11.82 as in original;

HELP @ 1.051; !residual variance of HELP set to .98;

PDA @ 133.023; !residual variance of PDA set to .954;

HELP on ILC @ 0.528; !a path;

PDA on HELP @ 3.239 ILC @ 0.522; !b path, c' path;

MODEL: !MODEL statement repeats all paths;

[HELP @ 0.744]; !mean of help set to 0 as in original;

[PDA @ 11.822]; !mean of pda set to 11.82 as in original;

HELP @ 1.051; !residual variance of HELP set to .98;

PDA @ 133.023; !residual variance of PDA set to .954;

HELP on ILC * 0.528;

PDA on HELP * 3.239 ILC * 0.522;

MODEL INDIRECT: !MODEL INDIRECT statement to obtain mediated ef

PDA IND ILC; !Start and endpoint of mediation model defi

*** WARNING

Input line exceeded 90 characters. Some input may be truncated.

cutpoints = ILC (0); !Define cutpoint to generate binary treatment;

*** WARNING

Input line exceeded 90 characters. Some input may be truncated.

MODEL INDIRECT: !MODEL INDIRECT statement to obtain mediated eff

*** WARNING

Input line exceeded 90 characters. Some input may be truncated.

PDA IND ILC; !Start and endpoint of mediation model defin

*** WARNING in ANALYSIS command

Starting with Version 5, TYPE=MEANSTRUCTURE is the default for all

analyses. To remove means from the model, use

MODEL=NOMEANSTRUCTURE in the ANALYSIS command.

4 WARNING(S) FOUND IN THE INPUT INSTRUCTIONS

MONTE CARLO POWER EXAMPLE 1 - MEDIATION PATH MODEL

SUMMARY OF ANALYSIS

Number of groups 1

Number of observations 124

Number of replications

Requested 10000

Completed 10000

Value of seed 2222

Number of dependent variables 2

Number of independent variables 1

Number of continuous latent variables 0

Observed dependent variables

Continuous

HELP PDA

Observed independent variables

ILC

Estimator ML

Information matrix OBSERVED

Maximum number of iterations 1000

Convergence criterion 0.500D-04

Maximum number of steepest descent iterations 20

SAMPLE STATISTICS FOR THE FIRST REPLICATION

SAMPLE STATISTICS

Means

HELP PDA ILC

________ ________ ________

1 0.982 16.243 0.613

Covariances

HELP PDA ILC

________ ________ ________

HELP 0.765

PDA 1.676 133.829

ILC 0.074 0.512 0.237

Correlations

HELP PDA ILC

________ ________ ________

HELP 1.000

PDA 0.166 1.000

ILC 0.173 0.091 1.000

MODEL FIT INFORMATION

Number of Free Parameters 3

Loglikelihood

H0 Value

Mean -656.889

Std Dev 11.035

Number of successful computations 10000

Proportions Percentiles

Expected Observed Expected Observed

0.990 0.988 -682.561 -683.356

0.980 0.976 -679.553 -680.168

0.950 0.946 -675.041 -675.548

0.900 0.899 -671.032 -671.117

0.800 0.800 -666.177 -666.195

0.700 0.708 -662.676 -662.477

0.500 0.507 -656.889 -656.696

0.300 0.305 -651.102 -650.933

0.200 0.202 -647.602 -647.533

0.100 0.098 -642.746 -642.852

0.050 0.046 -638.737 -639.174

0.020 0.016 -634.226 -635.041

0.010 0.007 -631.218 -632.441

H1 Value

Mean -654.848

Std Dev 11.242

Number of successful computations 10000

Proportions Percentiles

Expected Observed Expected Observed

0.990 0.993 -681.000 -679.536

0.980 0.984 -677.935 -677.141

0.950 0.952 -673.339 -673.061

0.900 0.904 -669.255 -669.000

0.800 0.799 -664.309 -664.360

0.700 0.694 -660.743 -660.970

0.500 0.491 -654.848 -655.132

0.300 0.294 -648.952 -649.165

0.200 0.202 -645.386 -645.331

0.100 0.103 -640.440 -640.248

0.050 0.054 -636.356 -635.976

0.020 0.024 -631.760 -630.969

0.010 0.013 -628.696 -627.764

Information Criteria

Akaike (AIC)

Mean 1319.779

Std Dev 22.071

Number of successful computations 10000

Proportions Percentiles

Expected Observed Expected Observed

0.990 0.993 1268.436 1270.850

0.980 0.984 1274.452 1276.031

0.950 0.954 1283.475 1284.341

0.900 0.902 1291.493 1291.675

0.800 0.798 1301.204 1301.042

0.700 0.695 1308.205 1307.862

0.500 0.493 1319.779 1319.373

0.300 0.292 1331.352 1330.953

0.200 0.200 1338.353 1338.355

0.100 0.101 1348.064 1348.229

0.050 0.054 1356.083 1357.091

0.020 0.024 1365.105 1366.315

0.010 0.012 1371.121 1372.688

Bayesian (BIC)

Mean 1328.239

Std Dev 22.071

Number of successful computations 10000

Proportions Percentiles

Expected Observed Expected Observed

0.990 0.993 1276.897 1279.311

0.980 0.984 1282.913 1284.492

0.950 0.954 1291.936 1292.802

0.900 0.902 1299.954 1300.135

0.800 0.798 1309.665 1309.503

0.700 0.695 1316.666 1316.323

0.500 0.493 1328.239 1327.833

0.300 0.292 1339.813 1339.413

0.200 0.200 1346.814 1346.816

0.100 0.101 1356.525 1356.690

0.050 0.054 1364.543 1365.552

0.020 0.024 1373.566 1374.776

0.010 0.012 1379.582 1381.149

Sample-Size Adjusted BIC (n* = (n + 2) / 24)

Mean 1318.753

Std Dev 22.071

Number of successful computations 10000

Proportions Percentiles

Expected Observed Expected Observed

0.990 0.993 1267.411 1269.825

0.980 0.984 1273.427 1275.006

0.950 0.954 1282.449 1283.316

0.900 0.902 1290.468 1290.649

0.800 0.798 1300.179 1300.017

0.700 0.695 1307.180 1306.837

0.500 0.493 1318.753 1318.347

0.300 0.292 1330.327 1329.927

0.200 0.200 1337.328 1337.330

0.100 0.101 1347.039 1347.204

0.050 0.054 1355.057 1356.066

0.020 0.024 1364.080 1365.290

0.010 0.012 1370.096 1371.663

Chi-Square Test of Model Fit

Degrees of freedom 4

Mean 4.083

Std Dev 2.889

Number of successful computations 10000

Proportions Percentiles

Expected Observed Expected Observed

0.990 0.989 0.297 0.288

0.980 0.979 0.429 0.418

0.950 0.952 0.711 0.718

0.900 0.903 1.064 1.082

0.800 0.808 1.649 1.698

0.700 0.707 2.195 2.225

0.500 0.512 3.357 3.435

0.300 0.309 4.878 4.960

0.200 0.209 5.989 6.105

0.100 0.109 7.779 7.975

0.050 0.057 9.488 9.813

0.020 0.022 11.668 11.877

0.010 0.011 13.277 13.542

RMSEA (Root Mean Square Error Of Approximation)

Mean 0.028

Std Dev 0.039

Number of successful computations 10000

Proportions Percentiles

Expected Observed Expected Observed

0.990 1.000 -0.063 0.000

0.980 1.000 -0.053 0.000

0.950 1.000 -0.037 0.000

0.900 1.000 -0.023 0.000

0.800 1.000 -0.005 0.000

0.700 0.412 0.007 0.000

0.500 0.366 0.028 0.000

0.300 0.280 0.048 0.044

0.200 0.221 0.061 0.065

0.100 0.143 0.078 0.090

0.050 0.092 0.092 0.108

0.020 0.050 0.108 0.126

0.010 0.029 0.119 0.139

SRMR (Standardized Root Mean Square Residual)

Mean 0.078

Std Dev 0.035

Number of successful computations 10000

Proportions Percentiles

Expected Observed Expected Observed

0.990 1.000 -0.003 0.020

0.980 1.000 0.007 0.024

0.950 0.988 0.021 0.031

0.900 0.939 0.033 0.039

0.800 0.804 0.049 0.049

0.700 0.668 0.060 0.057

0.500 0.434 0.078 0.073

0.300 0.255 0.096 0.091

0.200 0.177 0.107 0.103

0.100 0.104 0.123 0.123

0.050 0.067 0.135 0.143

0.020 0.039 0.149 0.165

0.010 0.026 0.159 0.186

MODEL RESULTS

ESTIMATES S. E. M. S. E. 95% % Sig

Population Average Std. Dev. Average Cover Coeff

HELP ON

ILC 0.528 0.5291 0.1302 0.1306 0.0170 0.951 0.980

PDA ON

HELP 3.239 3.2476 0.9121 0.9076 0.8319 0.948 0.944

ILC 0.522 0.5256 1.8857 1.8727 3.5556 0.947 0.062

Intercepts

HELP 0.744 0.7440 0.0000 0.0000 0.0000 1.000 0.000

PDA 11.822 11.8220 0.0000 0.0000 0.0000 1.000 0.000

Residual Variances

HELP 1.051 1.0510 0.0000 0.0000 0.0000 1.000 0.000

PDA 133.023 133.0230 0.0000 0.0000 0.0000 1.000 0.000

QUALITY OF NUMERICAL RESULTS

Average Condition Number for the Information Matrix 0.461E-02

(ratio of smallest to largest eigenvalue)

TOTAL, TOTAL INDIRECT, SPECIFIC INDIRECT, AND DIRECT EFFECTS

ESTIMATES S. E. M. S. E. 95% % Sig

Population Average Std. Dev. Average Cover Coeff

Effects from ILC to PDA

Total 2.232 2.2440 1.6904 1.6762 2.8572 0.947 0.275

Tot indirect 1.710 1.7184 0.6515 0.6522 0.4244 0.932 0.853

Specific indirect

PDA

HELP

ILC 1.710 1.7184 0.6515 0.6522 0.4244 0.932 0.853

Direct

PDA

ILC 0.522 0.5256 1.8857 1.8727 3.5556 0.947 0.062

TECHNICAL OUTPUT

PARAMETER SPECIFICATION

NU

HELP PDA ILC

________ ________ ________

1 0 0 0

LAMBDA

HELP PDA ILC

________ ________ ________

HELP 0 0 0

PDA 0 0 0

ILC 0 0 0

THETA

HELP PDA ILC

________ ________ ________

HELP 0

PDA 0 0

ILC 0 0 0

ALPHA

HELP PDA ILC

________ ________ ________

1 0 0 0

BETA

HELP PDA ILC

________ ________ ________

HELP 0 0 1

PDA 2 0 3

ILC 0 0 0

PSI

HELP PDA ILC

________ ________ ________

HELP 0

PDA 0 0

ILC 0 0 0

STARTING VALUES

NU

HELP PDA ILC

________ ________ ________

1 0.000 0.000 0.000

LAMBDA

HELP PDA ILC

________ ________ ________

HELP 1.000 0.000 0.000

PDA 0.000 1.000 0.000

ILC 0.000 0.000 1.000

THETA

HELP PDA ILC

________ ________ ________

HELP 0.000

PDA 0.000 0.000

ILC 0.000 0.000 0.000

ALPHA

HELP PDA ILC

________ ________ ________

1 0.744 11.822 0.613

BETA

HELP PDA ILC

________ ________ ________

HELP 0.000 0.000 0.528

PDA 3.239 0.000 0.522

ILC 0.000 0.000 0.000

PSI

HELP PDA ILC

________ ________ ________

HELP 1.051

PDA 0.000 133.023

ILC 0.000 0.000 0.237

POPULATION VALUES

NU

HELP PDA ILC

________ ________ ________

1 0.000 0.000 0.000

LAMBDA

HELP PDA ILC

________ ________ ________

HELP 1.000 0.000 0.000

PDA 0.000 1.000 0.000

ILC 0.000 0.000 1.000

THETA

HELP PDA ILC

________ ________ ________

HELP 0.000

PDA 0.000 0.000

ILC 0.000 0.000 0.000

ALPHA

HELP PDA ILC

________ ________ ________

1 0.744 11.822 0.000

BETA

HELP PDA ILC

________ ________ ________

HELP 0.000 0.000 0.528

PDA 3.239 0.000 0.522

ILC 0.000 0.000 0.000

PSI

HELP PDA ILC

________ ________ ________

HELP 1.051

PDA 0.000 133.023

ILC 0.000 0.000 0.250

DIAGRAM INFORMATION

Mplus diagrams are currently not available for Monte Carlo analysis.

No diagram output was produced.

Beginning Time: 13:29:11

Ending Time: 13:29:20

Elapsed Time: 00:00:09

MUTHEN & MUTHEN

3463 Stoner Ave.

Los Angeles, CA 90066

Tel: (310) 391-9971

Fax: (310) 391-8971

Web: www.StatModel.com

Support: Support@StatModel.com

Copyright (c) 1998-2015 Muthen & Muthen
